# Integrative Physiological and Transcriptome Analysis Reveals the Mechanism of Cd Tolerance in *Sinapis alba*

**DOI:** 10.3390/genes14122224

**Published:** 2023-12-16

**Authors:** Mengxian Cai, Tinghai Yang, Shiting Fang, Lvlan Ye, Lei Gu, Hongcheng Wang, Xuye Du, Bin Zhu, Tuo Zeng, Tao Peng

**Affiliations:** School of Life Sciences, Guizhou Normal University, Guiyang 550025, China; 201706002@gznu.edu.com (M.C.); 222100100455@gznu.edu.cn (T.Y.); 232100100391@gznu.edu.cn (S.F.); 222100100456@gznu.edu.cn (L.Y.); 201808009@gznu.edu.cn (L.G.); wanghc@gznu.edu.cn (H.W.); duxuye@gznu.edu.cn (X.D.); zhugg130@126.com (B.Z.); zengtuo@gznu.edu.cn (T.Z.)

**Keywords:** *Sinapis alba*, transcriptome, Cd stress, physiological response

## Abstract

Recently, pollution caused by the heavy metal Cd has seriously affected the environment and agricultural crops. While *Sinapis alba* is known for its edible and medicinal value, its tolerance to Cd and molecular response mechanism remain unknown. This study aimed to analyze the tolerance of *S. alba* to Cd and investigate its molecular response mechanism through transcriptomic and physiological indicators. To achieve this, *S. alba* seedlings were treated with different concentrations of CdCl_2_ (0.25 mmol/L, 0.5 mmol/L, and 1.0 mmol/L) for three days. Based on seedling performance, *S. alba* exhibited some tolerance to a low concentration of Cd stress (0.25 mmol/L CdCl_2_) and a strong Cd accumulation ability in its roots. The activities and contents of several antioxidant enzymes generally exhibited an increase under the treatment of 0.25 mmol/L CdCl_2_ but decreased under the treatment of higher CdCl_2_ concentrations. In particular, the proline (Pro) content was extremely elevated under the 0.25 and 0.5 mmol/L CdCl_2_ treatments but sharply declined under the 1.0 mmol/L CdCl_2_ treatment, suggesting that Pro is involved in the tolerance of *S. alba* to low concentration of Cd stress. In addition, RNA sequencing was utilized to analyze the gene expression profiles of *S. alba* exposed to Cd (under the treatment of 0.25 mmol/L CdCl_2_). The results indicate that roots were more susceptible to disturbance from Cd stress, as evidenced by the detection of 542 differentially expressed genes (DEGs) in roots compared to only 37 DEGs in leaves. GO and KEGG analyses found that the DEGs induced by Cd stress were primarily enriched in metabolic pathways, plant hormone signal transduction, and the biosynthesis of secondary metabolites. The key pathway hub genes were mainly associated with intracellular ion transport and cell wall synthesis. These findings suggest that *S. alba* is tolerant to a degree of Cd stress, but is also susceptible to the toxic effects of Cd. Furthermore, these results provide a theoretical basis for understanding Cd tolerance in *S. alba*.

## 1. Introduction

With developing industrialization and urbanization, heavy metal pollutants, including Cd, Cu, Pb, and Mn, have become a severe issue in soil [1], because excess heavy metal levels in soil not only destroy the function of soil and inhibit the growth and development of plants and soil microorganisms that are intolerant to heavy metals, but also cause serious toxicity to crops and cash crops, resulting in a serious decline in crop yields [2,3]. Moreover, heavy metals can be transported along the food chain and negatively impact human health. Among these heavy metal pollutants, Cd pollution is the most globally distributed and causes the most serious threat to crops because Cd has very high mobility in soil-plant systems, leading to the accumulation of Cd in crops. Thus, determining the Cd tolerance of crops and revealing the tolerance mechanism to Cd in these crops have become a priority [4,5].

Excessive Cd in plants has been found to have a negative impact on various aspects of plant physiology. It not only indirectly affects the synthesis and decomposition of sugar, protein, and fat, but also interferes with plant nutrition, hormone metabolism, and other essential activities. As a result, there is a shortage of substances and energy needed for plant growth, resulting in inhibited plant growth [6,7]. In addition, Cd has been found to induce the production of high levels of reactive oxygen species (ROS) in plants, which can impede growth, development, and reproduction. Moreover, excess ROS production can significantly increase lipid peroxidation, potentially leading to cell death [8,9]. The entry of heavy metals into plants also destroys the internal structure of chloroplasts, negatively impacting nutrient uptake, transfer, and chloroplast synthesis [10].

To reduce the levels of ROS caused by stresses, plants produce a variety of antioxidant enzymes, including superoxide dismutase (SOD), peroxidase (POD), ascorbate peroxidase (APX), and catalase (CAT). These enzymes are crucial for regulating osmotic pressure in the plant cytoplasm following abiotic stress. They also help stabilize biomolecular structures, decrease cellular acidity, and regulate cellular redox [11,12]. By converting excessive reactive oxygen species and free radicals into less toxic substances, antioxidant enzymes help maintain a balanced level of ROS in plant cells and provide protection against heavy metal stress [13,14]. There is a positive correlation between proline accumulation and plant tolerance to various abiotic stresses. Because of its metal-chelating properties, proline (Pro) acts as a molecular chaperone, an antioxidant defense molecule that clears ROS, and has signal transduction behavior that activates specific gene functions, which is critical for plant recovery from stress [15]. In addition, recent studies have shown that certain transcription factors (TFs) play a role in plant responses to abiotic stress [16]. For instance, some *WRKY* genes have been found to enhance plant tolerance to Cd or maintain metal ion homeostasis by regulating downstream functional genes. *AtWRKY12* inhibits the expression of genes involved in plant chelator protein synthesis by directly binding to the W-box of the *GSH1* promoter, indirectly repressing the expression of genes related to chelator protein synthesis in plants. However, it negatively regulates Cd tolerance in *Arabidopsis thaliana* [17]. Another TF, MYB49, can directly bind to the promoters of *bHLH38* and *bHLH101*, leading to the activation of IRT1, which is involved in the uptake of Cd. *MYB49* also binds to the promoter regions of heavy-metal-associated *HIPP22* and *HIPP44*, resulting in an up-regulation of their expression and subsequently increasing Cd accumulation [18].

*S. alba* (2n = 24) is a versatile plant with various medicinal and food uses. It serves as an edible oil, edible vegetable, condiment, fodder and green manure source [19]. In Europe, *S. alba* is well-known as the primary mustard variety. Recent research indicates that *S. alba* has potential applications as biodiesel. Additionally, *S. alba* rapeseed oil is utilized as a lubricant, while the seeds of *S. alba* are used in traditional medicine for their antitumor and antiviral effects [20,21]. These applications significantly enhance the medicinal and economic value of *S. alba*. Notably, *S. alba* exhibits remarkable tolerance to drought and high temperatures, making it particularly suitable for cultivation in arid regions. A high-quality genome sequence of *S. alba* has been released recently, affording a facility for its molecular analysis. However, its tolerance to Cd and the underlying molecular response mechanism remain elusive. In this study, we utilized phenotypic, physiological, and comparative transcriptomic analyses to investigate the Cd accumulation capability as well as the physiological and transcriptomic responses of *S. alba* under Cd stress. Our findings indicate that *S. alba* exhibits tolerance to low-concentration Cd stress. As a secondary amine, the content of proline was significantly increased under Cd stress, indicating that the degree of abiotic stress on plants was different. The differentially expressed genes (DEGs) induced by Cd stress were primarily enriched in metabolic pathways, plant hormone signal transduction (jasmonic acid and auxin), and the biosynthesis of secondary metabolites. Furthermore, our study revealed the presence of several transcription factors (TFs) associated with Cd tolerance, as well as the identification of two crucial DEGs involved in cell ion absorption and transporters: *WRKY70* (*Sal11g08240L*) and *MYB40* (*Sal12g05270L*). These findings provide valuable insights into the molecular mechanism and physiological regulation of Cd tolerance in *S. alba*. This study also establishes a theoretical foundation for the utilization of *S. alba* in food processing and medicine.

## 2. Materials and Methods

### 2.1. Seedlings Treatment and Experimental Conditions

A pure line of *S. alba* (accession number: BJ023) of a good seed yield was used in this study. The *S. alba* seeds were germinated in a sterile culture dish (9 cm in diameter). To enhance the germination rate of the seeds, two layers of qualitative filter paper were placed at the bottom of the petri dish to retain moisture. Additionally, a 1/4 Hoagland nutrient solution was replaced daily throughout this period. After one week, the seedlings were transferred to a black culture box filled with vermiculite (a non-nutritive soil) for duration of 14 days. During this time, irrigation was performed daily using a 1/2 Hoagland nutrient solution. After 14 days, the plants were divided into 4 groups, with 48 plants in each group. Anhydrous CdCl_2_ was dissolved in 1/2 Hoagland solution to prepare different concentrations of Cd solution (0.25 mmol/L, 0.5 mmol/L, and 1.0 mmol/L). The amount of each solution added was 1 L. The CK group was irrigated with the same amount of 1/2 Hoagland nutrient solution. The solution was changed once a day. After 3 days of treatment, through the measurement of its physiological indexes in the early stage, it was observed that exposure to a 0.25 mmol/L Cd solution for 3 days resulted in a significant increase in most antioxidant enzymes in the seedlings, with no obvious phenotypic changes. However, when treated with other concentrations of Cd solution for 3 days, most antioxidant enzymes in the body decreased significantly, and the phenotypic changes become more pronounced. The main objective of this study was to investigate the tolerance of *S. alba* to Cd. Therefore, in this study, the roots and leaves of *S. alba* treated with 0.25 mmol/L were selected for RNA-seq sequencing. The physiological indexes of the shoots of *S. alba* were measured after exposure to different concentrations of Cd (0 mmol/L, 0.25 mmol/L, 0.5 mmol/L, and 1 mmol/L). The experiment was conducted in the tissue culture room of the School of Life Sciences at Guizhou Normal University. The temperature was maintained at a constant 19℃ throughout the day and night, with a 12 h light and dark cycle. The light intensity was set at 7000 Lux, and the relative humidity was maintained at 60%.

### 2.2. Determination of Cd Content in Roots and Shoots of S. alba

After three days of treatment with different concentrations of Cd, the Cd content in roots and shoots of *S. alba* was determined. First, it was washed three times with tap water for ten minutes. Then, it was soaked in sterile deionized water for 20 min, and excess water was removed using absorbent paper. The tested *S. alba* plants were divided into shoots and roots. Both parts were dried in an oven at 37 °C. Then, 0.25 g of the dried sample was weighed and placed into the digestion tank. To this, 8 mL of concentrated nitric acid and 2 mL of perchloric acid were added. The solution was digested at a variable temperature of 100 °C in the digestion tank. After digestion, the solution was transferred to a 25 mL volumetric flask. The inner tank and cover were washed with a small amount of 1% nitric acid solution, ensuring at least 3 washing cycles. The solution was then pipetted into a volumetric flask using a glass rod and diluted to the mark with 1% nitric acid. Filtration was performed using a filter membrane and syringe, while a reagent blank experiment was conducted simultaneously. The standard curve was drawn using a mixed standard, and the content of Cd in *S. alba* was detected using AAS (atomic absorption spectrometry).

### 2.3. Determination of Physiological Indexes

The physiological indicators followed the methodology described by Zuo et al. [22]. To determine the chlorophyll content, approximately 0.1 g of leaves was weighed and placed into a mortar. Then, 1 mL of extract (acetone:anhydrous ethanol = 2:1) and 10 mg of reagent one (chlorophyll content detection kit) were added, and the mixture was ground in dark conditions. The chlorophyll content in the leaves was subsequently determined according to the instructions provided by the chlorophyll content detection kit (Solarbio, Beijing, China). For the determination of proline content, approximately 0.1 g of leaves were weighed and placed into a mortar, and 1 mL of extract (proline content detection kit) was added for grinding. The proline content in the leaves was then determined following the instructions provided by the proline content detection kit (Solarbio, Beijing, China). The determination of MDA content involved weighing approximately 0.1 g of leaves and placing them into a mortar. Then, 1 mL of extract (MDA content detection kit) was added and fully ground. After centrifugation for 10 min, the supernatant was collected and placed on ice. The MDA content in the leaves was determined following the instructions provided in the MDA content detection kit (Solarbio, Beijing, China). Superoxide dismutase (SOD), ascorbate peroxidase (APX), catalase (CAT), and peroxidase (POD) were also detected using the instructions provided in the kit (Solarbio, Beijing, China). Each group consisted of a control group and an experimental group, with three parallel control groups in each.

### 2.4. RNA Extraction, cDNA Library Preparation, and Transcriptome Sequencing

The roots and shoots from seedlings of *S. alba* treated with 0.25 mmol/L CdCl_2_ for 3 days were collected, and the total RNA was extracted using a commercial plant RNA extraction kit (Solarbio, Beijing, China). Three repeats per samples were prepared in this study. To confirm the quality of the extracted RNA, the concentration of RNA and RNA integrity were detected using a microplate reader and agarose gel electrophoresis (AGE). The qualified RNA was sent to Biomarker Technologies (Beijing, China) for RNA-seq sequencing. Then the *c*DNA libraries were obtained following the TruSeq RNA Sample Prep v2 protocol (Illumina, San Diego, CA, USA). In total, 12 *c*DNA libraries were constructed and subjected to sequencing using the Illumina NovaSeq 6000 platform.

The raw data from RNA-seq were filtered using fastp (v0.20.1) with default parameters [23]. Trimmomatic version 0.33 was utilized to obtain clean reads by removing reads containing adapters, poly-N, and low-quality bases. Subsequently, these clean reads were mapped to the recently released reference gene of *S. alba* [24,25] using Hisat2 (v2.2.1) with default settings. The transcript assembly was performed using StringTie (v2.2.1) software [18]. The FPKM (fragments per kilobase per million bases) value of the gene was calculated using DESeq2 (v1.36.0) [26]. DESeq2 (v1.36.0) was used to determine differentially expressed genes under the threshold of padj < 0.05 and |log2FoldChange| > 1. GO (Gene Ontology) enrichment and KEGG (Kyoto Encyclopedia of Genes and Genomes) enrichment analyses of the DEGs were performed using OmicShare cloud platform tools (https://www.omicshare.com/tools, accessed on 30 August 2023). The raw sequence data are available in NCBI-SRA (https://www.ncbi.nlm.nih.gov/sra, accessed on 6 September 2021) under accession number PRJNA760315.

### 2.5. Verification of RNA-Seq Results

The expression levels of DEGs in RNA-seq sequencing were validated using qRT-PCR analysis. For this verification, five up-regulated and five down-regulated genes were randomly selected. The actin gene (*Sal05g04640L*) of *S. alba* was used as the internal reference gene control in this study. A 20 μL qRT-PCR mixture was prepared using SYBR-GREEN fluorescent reagents (Solarbio, Beijing, China). The mixture consisted of 2 μL *c*DNA template, 10 μL 2xSYBR Green RT-PCR (RT-qPCR) Mix, 0.4 μL 10 μM Primer Forward, 0.4 μL 10 μM Primer Reverse, and 7.2 μL ddH20. The primers of the selected genes were designed using NCBI primer-blast technology (Table S2).

### 2.6. Data Analysis

The significance of all data was determined via Excel and IBM SPSS Statistics 26 (SPSS Inc., Chicago, IL, USA) under the cutoff of *p* < 0.05. The multiple comparisons were conducted using the LSD method (*p* < 0.05). The error bar in chart indicates the SD, and the experiment was repeated three times (*n* = 3).

## 3. Results

### 3.1. Cd Tolerance and Accumulation in S. alba

**Figure 1:**
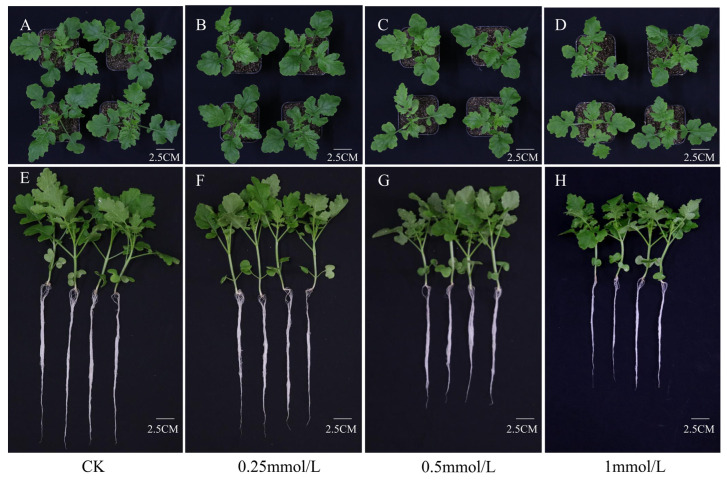
Seedlings performance of *S. alba* treated with different concentrations of CdCl_2_ solutions. (**A**–**D**): the performance of shoots of *S. alba* plants treated with 0 mmol/L (CK), 0.25 mmol/L, 0.5 mmol/L, and 1 mmol/L CdCl_2_ solutions for 3 days. (**E**–**H**): the performance of young seedlings treated with 0 mmol/L (CK), 0.25 mmol/L, 0.5 mmol/L, and 1 mmol/L CdCl_2_ solutions for 3 days.

**Figure 2:**
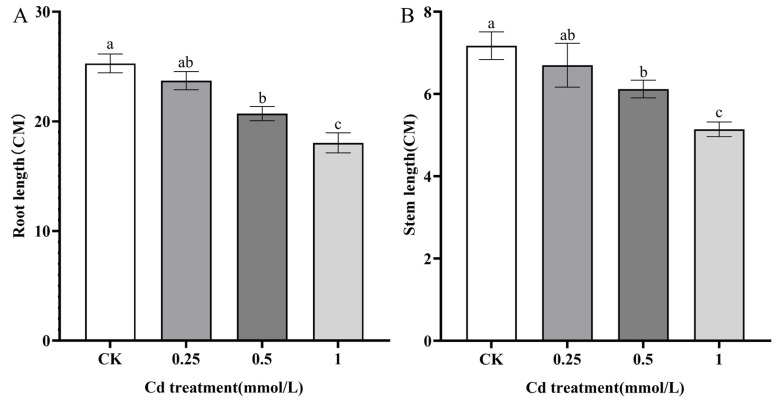
The changes in root and stem length of *S. alba* after treatment with different concentrations of CdCl_2_. (**A**): root length. (**B**): stem length. Different letters represent the significantly different groups, and significance was determined by multiple comparisons using Fisher’s least significant difference (LSD) method (*p* < 0.05). The error bar in chart indicates standard deviation (SD), and three replicates (*n* = 3) per sample are used to calculate the value of mean ± SD.

The *S. alba* seedlings were incubated for 2 weeks in 1/2 Hoagland solution and then treated with different concentrations (0.25 mmol/L, 0.5 mmol/L, and 1 mmol/L) of CdCl_2_ solution for 3 days. Compared with the CK, there was no significant change in shoots and roots under the 0.25 mmol/L CdCl_2_ treatment (Figure 1), but seedling performance was seriously affected under 0.5 and 1.0 mmol/L CdCl_2_ treatments. In addition, the root length and stem length of seedlings under 0.25 mmol/L CdCl_2_ were comparable to those of CK. Under the conditions of 0.5 and 1.0 mmol/L CdCl_2_, they all decreased significantly (Figure 2), indicating that birch was tolerant to low concentrations of Cd stress.

**Figure 3:**
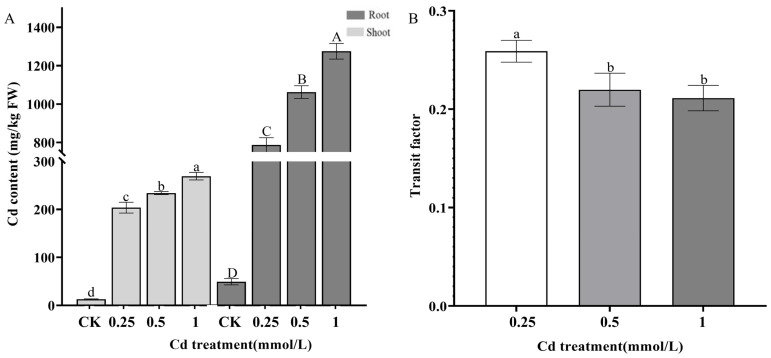
Cd accumulation and transport factors in *S. alba* under different Cd treatments. (**A**): Cd content. (**B**): Cd transport factor in different treatments. Different letters represent the significantly different groups, which were determined by multiple comparisons using Fisher’s least significant difference (LSD) method (*p* < 0.05). The error bar in the chart indicates the standard deviation (SD), and three replicates (*n* = 3) per sample were used to calculate the mean ± SD.

To investigate the ability of Cd accumulation in *S. alba*, the atomic absorption spectroscopy method was used to determine the Cd content in *S. alba* after 3 days of treatment with CdCl_2_ (Figure 3A; Table S1). The results show that the Cd content in dehydrated shoots and roots of *S. alba* was significantly elevated (LSD-test, *p* < 0.01) with the increasing concentrations of CdCl_2_. In brief, the Cd contents in dehydrated shoots were 203.59 ± 5.20 mg/kg, 233.88 ± 3.68 mg/kg, and 269.25 ± 2.88 mg/kg under the treatment with 0.25 mmol/L, 0.5 mmol/L, and 1.0 mmol/L CdCl_2_, respectively. Correspondingly, the Cd contents in dehydrated roots were 786.99 ± 21.45 mg/kg, 1065.29 ± 31.30 mg/kg, and 1275.42 ± 39.75 mg/kg. However, the analysis of the transport coefficient (the ratio of the content of elements in the aboveground part to the content of the same element in the underground part) of Cd from *S. alba* showed that under the treatment of 0.25 mmol/L, 0.5 mmol/L, and 1 mmol/L CdCl_2_ (Figure 3B), the transport coefficient of *S. alba* Cd was less than 0.5. This result shows that *S. alba* has a weak ability to transport Cd from the underground part to the aboveground part, which is not conducive to the recovery and utilization of heavy metals.

### 3.2. Physiological Responses to Cd in S. alba

**Figure 4:**
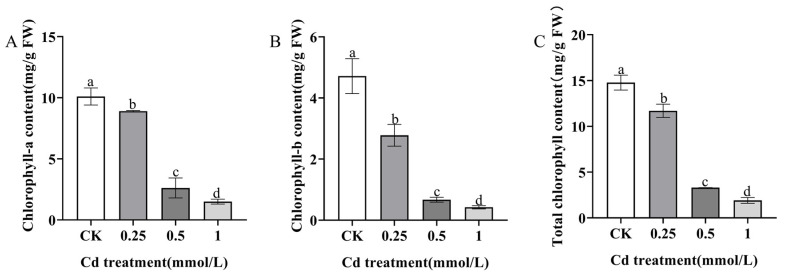
Chlorophyll content changes in *S. alba* seedlings under different concentrations of Cd treatment. (**A**): chlorophyll-a content. (**B**): chlorophyll-b content. (**C**): total chlorophyll content. Different letters represent significantly different groups determined by the LSD method (*p* < 0.05). The error bar in the chart indicates the standard deviation (SD), and three replicates (*n* = 3) per sample were used to calculate the mean ± SD.

**Figure 5:**
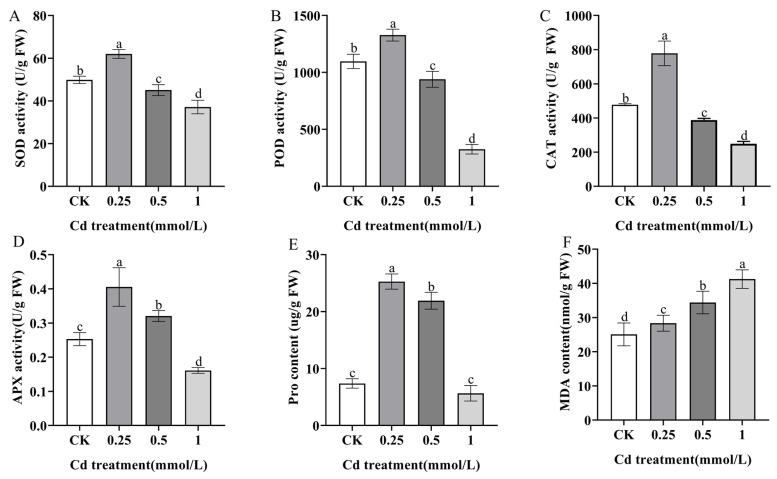
Physiological indicators change in *S. alba* seedlings under different concentrations of Cd treatment. (**A**): SOD activity. (**B**): POD activity. (**C**): CAT activity. (**D**): APX activity. (**E**): Pro content. (**F**): MDA content. Different letters represent significantly different groups determined via the LSD method (*p* < 0.05). The error bar in the chart indicates the standard deviation (SD), and three replicates (*n* = 3) per sample were used to calculate the mean ± SD.

Some physiological indicators, including chlorophyll, SOD, APX, CAT, MDA, Pro, and POD, were employed to ascertain the extent of physiological responses to Cd stress in *S. alba*. *S. alba* exhibited a significant reduction in chlorophyll a, chlorophyll b, and total chlorophyll levels when subjected to different concentrations of Cd stress (Figure 4). The decrease in chlorophyll content was particularly notable at Cd concentrations of 0.5 mmol/L and 1 mmol/L. Compared to the CK, the activities of SOD, POD, CAT, and APX were significantly induced (*p* < 0.05) under the treatment with 0.25 mmol/L CdCl_2_, but obviously declined under the treatments with 0.5 mmol/L and 1.0 mmol/L CdCl_2_ (Figure 5A–D). We found that under 0.25 mmol/L and 0.5 mmol/L CdCl_2_ treatments, the content of Pro produced by plants under abiotic stress increased to threefold, while it decreased sharply under 1.0 mmol/L CdCl_2_ treatment (Figure 5E), indicating that when *S. alba* was subjected to Cd stress, Pro, as an important signal transduction factor, activated specific gene functions and is essential for plant recovery from stress. In addition, the MDA content in tested seedlings increased gradually with the Cd concentration (Figure 5F), suggesting that Cd caused membrane lipid peroxidation in *S. alba*.

### 3.3. Gene Expression Patterns Responding to Cd Stress in S. alba

To reveal the potential genes involved in the Cd tolerance in *S. alba*, we conducted a comparative transcriptome analysis to investigate the gene expression patterns of the control (CK) and CdCl_2_ treated seedlings (Cd). Only when treated with 0.25 mmol/L Cd solution for 3 days, most of the antioxidant enzymes in *S. alba* increased significantly, and the phenotypic changes were not obvious. Therefore, seedlings under the 0.25 mmol/L CdCl_2_ treatment were used to check the gene expression change. In total, 12 *c*DNA libraries of *S. alba* (6 libraries for roots and 6 libraries for shoots) were prepared. After trimming the adapters and removing the low-quality reads using FastQC with default parameters, 19,246,416–24,331,775 end-paired clean reads with a Q30 value > 94.34% (75.79 Gb clean data in total) were obtained from the 12 libraries. Then, the clean reads per sample were mapped to the recently released *S. alba* reference genome [24]. The fragments per kilobase per million mapped fragments value (FPKM) of the genes was employed to assess the gene expression profiles. To verify the accuracy of the RNA-seq data, 10 genes (5 up-regulated and 5 down-regulated genes) were randomly selected to determine the relative expression levels using a quantitative real-time polymerase chain reaction (qRT-PCR) (Table S2). The results of the qRT-PCR were consistent with those of RNA-seq analysis (Figure S1), confirming the reliability of the RNA-seq results. Compared to the CK, a total of 37 DEGs, comprising of 19 up-regulated and 18 down-regulated DEGs, were detected in shoots when exposed to Cd (CKs vs. Cds). In the comparison of roots of CK- and Cd-treated seedlings (CKr vs. Cdr), 542 DEGs (CKr vs. Cdr), including 137 up-regulated genes and 405 predominantly down-regulated genes (χ^2^-test, *p* < 0.001), were identified (Table S3). These results indicate that the genes in roots were likely prone to disturbance by Cd.

### 3.4. GO and KEGG Analyses of DEGs in Response to Cd Stress

**Figure 6:**
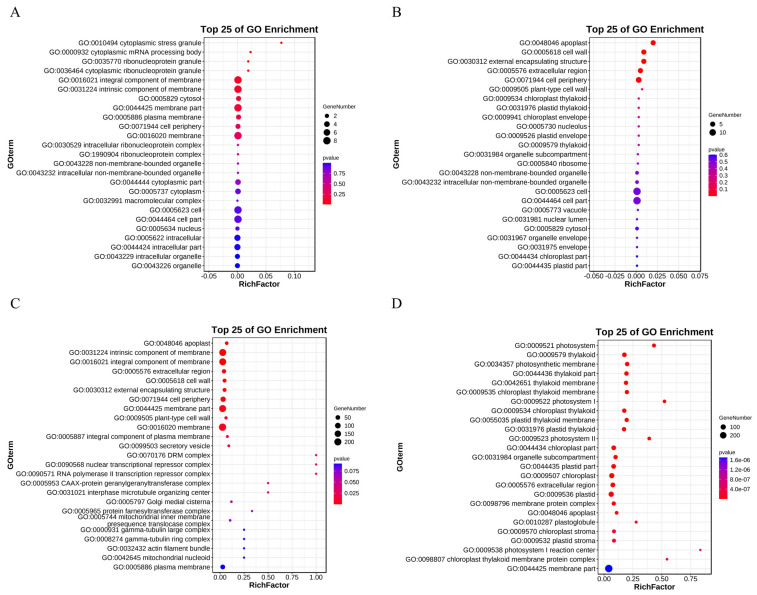
Gene ontology analysis of *S. alba* under Cd conditions. (**A**,**C**) Up-regulated DEGs enrichment in shoots and roots. (**B**,**D**) Enrichment of down-regulated DEGs in shoots and roots.

To investigate the role of DEGs after subjecting *S. alba* to Cd-induced stress, we conducted GO enrichment analysis on the identified DEGs. The analysis aimed to identify GO terms where the differential genes were significantly enriched compared to all annotated genes. The results of the GO enrichment analysis reveal significant enrichment of both up-regulated and down-regulated genes in biological process, cellular component, and molecular function categories in the comparisons of CKs vs. Cds and CKr vs. Cdr. The biological process section was mainly enriched in the categories of cellular process, single-organism process, and metabolic process. In the cellular component section, it was mainly enriched in the cell, cell part, membrane and membrane part categories. In the molecular function section, it was mainly enriched in the binding, catalytic activity, and nucleic acid binding transcription factor activity categories (Figure 6). The results suggest that *S. alba* may up-regulate the production of antioxidant compounds in response to plant oxidative stress caused by Cd stress [27].

**Figure 7:**
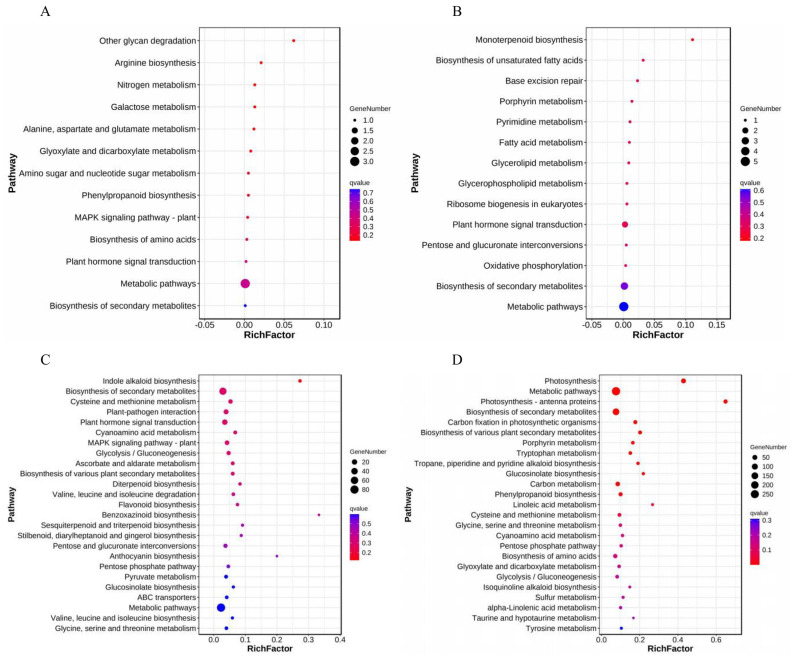
KEGG pathway enrichment analysis of significant DEGs. (**A**,**C**) Up-regulated DEG enrichment in shoots and roots. (**B**,**D**) Enrichment of down-regulated DEGs in shoots and roots.

To investigate the roles and effects of DEGs on the biological pathways of *S. alba* under Cd stress, Kyoto Encyclopedia of Genes and Genomes (KEGG) enrichment analysis was performed. The analysis revealed that the top five KEGG terms of up-regulated DEGs in CKs vs. Cds were involved in the metabolic pathway, biosynthesis of secondary metabolites, plant hormone signal transduction, biosynthesis of amino acids, and MAPK signaling pathway–plant categories (Figure 7A). The top five KEGG terms involved in down-regulated DEGs in CKs vs. Cds were found to be associated with metabolic pathways, biosynthesis of secondary metabolites, plant hormone signal transduction, oxidative phosphorylation, and pentose and glucuronate interconversions (Figure 7B). Similarly, the top five up-regulated differential genes in CKr vs. Cdr were also related to metabolic pathways, biosynthesis of secondary metabolites, plant hormone signal transduction, ribosome, and carbon metabolism (Figure 7C). The top five down-regulated DEGs enriched in KEGG pathways in CKr vs. Cdr were: “metabolic pathways”, biosynthesis of secondary metabolites, photosynthesis, carbon metabolism, and biosynthesis of amino acids (Figure 7D). The differential genes of CKs vs. Cds and CKr vs. Cdr were significantly enriched in the hormone and metabolic pathways. This suggests that *S. alba* alleviates the toxic effect of Cd mainly through metabolic pathways and hormone synthesis after Cd stress [28].

### 3.5. Involvement of DEGs from Underground Parts in Phytohormone Signalings

**Figure 8:**
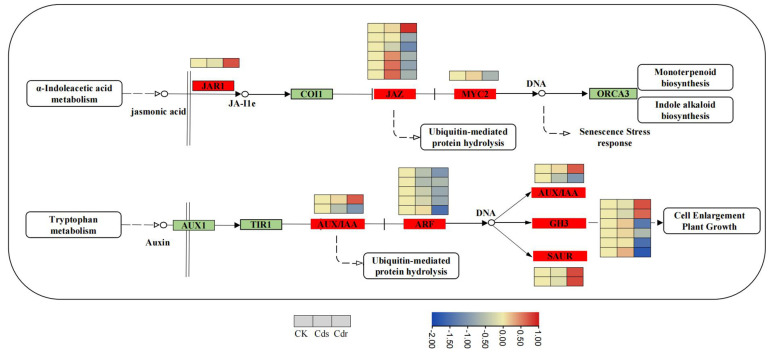
Transcriptional changes in DEGs involved in plant hormone biosynthesis and plant hormone signal transduction in shoots and roots. The heatmaps from left to right are CK, Cds, and Cdr in shoots and roots.

The functional analysis of the differentially expressed gene pathways revealed significant enrichment of DEGs in the plant hormone signal transduction pathway under Cd stress. Therefore, we conducted a detailed analysis of the DEGs related to this pathway, with special attention given to the 24 DEGs involved in the biosynthetic pathway of jasmonic acid and auxin primarily in the underground fraction (Figure 8, Table S4). In this study, we observed that of the total number of genes analyzed, 67% (16 genes) were down-regulated, while 33% (8 genes) were up-regulated. Interestingly, we identified eight DEGs that are directly associated with the biosynthesis of jasmonic acid. These DEGs included one gene encoding the JAR1 protein, one gene encoding the MYC2 protein, and six genes encoding the JAZ protein. A total of 16 DEGs were identified in the auxin biosynthesis pathway. Among these DEGs, five mainly encoded ARF proteins and seven mainly encoded *GH3* proteins. The findings suggest that Cd stress may significantly impact the growth and development of *S. alba*. Furthermore, the DEGs involved in the jasmonic acid and auxin signaling pathways were mostly down-regulated in roots.

### 3.6. Effect of Cd on Antioxidant Enzyme Activities of and Pro S. alba Cell Wall Synthesis, and Transcription Factor

**Figure 9:**
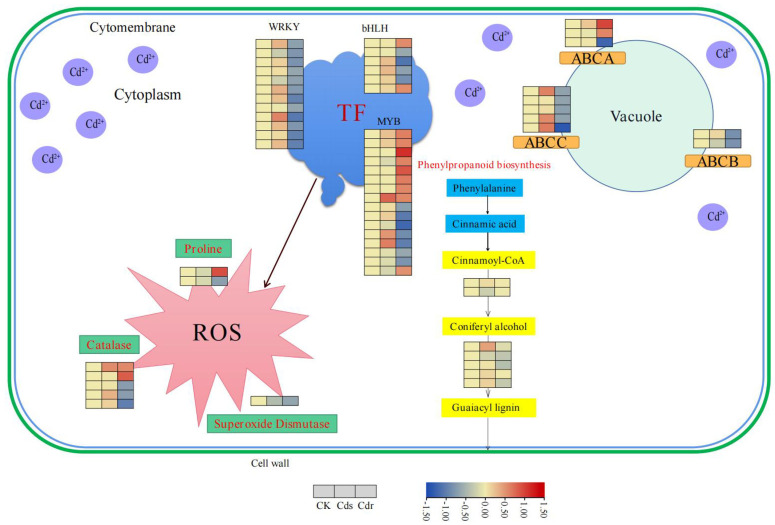
Transcriptome network in shoots and roots. The heatmaps from left to right are CK, Cds, and Cdr in shoots and roots.

Previous studies have demonstrated that SOD, CAT and Pro antioxidant enzymes have the ability to counteract the reactive oxygen species (ROS) produced during heavy metal stress by cleaning plant cells, thereby aiding in detoxification at the physiological level [29,30]. In our current investigation, we observed that *S. alba* exposed to Cd stress exhibited eight DEGs associated with the biosynthesis of SOD, CAT, and Pro antioxidant enzymes in the roots. Specifically, we identified one down-regulated DEG involved in SOD biosynthesis, two DEGs (one up-regulated and one down-regulated) involved in Pro biosynthesis, and five DEGs (three up-regulated and two down-regulated) involved in CAT biosynthesis (Figure 9, Table S5).

### 3.7. Analysis of TFs of S. alba during Cd Stress

Li et al. [31] demonstrated that transcription factors contain numerous phosphorylation sites, which play a crucial role in regulating heavy metal stress by controlling the expression of downstream genes. In this study, the response of *S. alba* to Cd stress resulted in the identification of 12 down-regulated DEGs from the *WRKY* transcription factor family, 16 DEGs from the *MYB* transcription factor family (9 up-regulated and 7 down-regulated), and 6 DEGs from the *bHLH* transcription factor family (2 up-regulated and 4 down-regulated) in the roots (Figure 9, Table S5).

### 3.8. Screening of DEGs Related to Transport and Absorption of Cd in S. alba

ABC transporter proteins are transmembrane transporter proteins that have important functions in phytohormone transport, lipid metabolism, detoxification of exogenous toxins, and plant stress resistance [32]. In this study, we identified a total of 10 DEGs in the roots, with 3 DEGs belonging to the ABCA transporter protein family, 1 DEG belonging to the ABCB transporter protein family, and 5 DEGs belonging to the ABCC transporter protein family (Figure 9, Table S5). In relation to the cell wall, the phenylpropanoid biosynthesis pathway is associated. In the roots, two DEGs (one up-regulated DEG and one down-regulated DEG) were found to be involved in the biosynthesis of Cinnamoyl-CoA in the phenylpropanoid biosynthesis pathway. Additionally, five DEGs (one up-regulated DEG and four down-regulated DEGs) were found to be associated with the biosynthesis of coniferyl alcohol (Figure 9, Table S6). Upon exposure to Cd stress, *S. alba* exhibited a significant down-regulated of DEGs involved in cell wall and antioxidant enzyme biosynthesis. This suggests that the plant has a low tolerance to Cd.

## 4. Discussion

When plants are exposed to Cd stress, an excessive amount of Cd can trigger the generation of excess ROS. These excess ROS can lead to lipid peroxidation, disruption of the biofilm system, and damage to proteins, carbohydrates, and other biological macromolecules [33]. Research has shown that plants possess an antioxidant enzyme system consisting of enzymes such as Pro, SOD, CAT, APX and POD. This system works synergistically to scavenge accumulated active oxygen free radicals under stress conditions, thereby reducing membrane lipid peroxidation and preventing heavy metal poisoning in plants [34,35,36,37].

Pro can form a complex with Cd^2+^ called Cd-Pro, which helps transport Cd^2+^ out of plant cells in vitro [38]. This reduces the toxic effect of Cd on plant cell bodies. Additionally, Pro also plays a crucial role in scavenging ROS induced by heavy metals in plants. It helps regulate cell osmotic pressure and redox [39]. SOD, the first antioxidant enzyme, plays a vital role in scavenging reactive oxygen species. It can rapidly convert superoxide anion radical to H_2_O_2_ and O^2^. Then, H_2_O_2_ is further converted to water and oxygen with the help of catalase (CAT), peroxidase, and the ascorbate-glutathione cycle system. Both SOD and APX are important in scavenging oxygen free radicals, preventing them from damaging the composition, structure, and function of cells. They play a crucial role in protecting cells from oxidative damage [40,41]. In this study, the effect of treating *S. alba* with different concentrations of CdCl_2_ on the activities of the three antioxidant enzymes (SOD, POD, and CAT) in the leaves was investigated. The results show that when the plants were treated with 0.25 mmol/L CdCl_2_ for three days, the activities of SOD, POD, and CAT increased significantly. However, as the concentration of CdCl_2_ increased to 0.5 mmol/L and 1 mmol/L, the activities of these enzymes decreased significantly, even lower than those in the control group. Additionally, compared to other antioxidant enzymes, APX activity and Pro content also decreased significantly at 0.5 mmol/L and 1 mmol/L. However, at 0.5 mmol/L, the APX activity and Pro content were still higher than those in the CK group (Figure 5). As one of the important protective enzymes in organisms, SOD can effectively scavenge oxygen free radicals. POD is a common oxidoreductase in plants that can prevent the accumulation of hydroxyl groups in plants. CAT is an enzyme scavenger and one of the key enzymes in the biological defense system. Under appropriate stress conditions, the synthesis of antioxidant enzymes in plants plays a role in promotion, but when it exceeds a certain limit, it is not conducive to the growth of plants, causing biological disorders. Exceeding the maximum tolerance range of the enzyme, the activity of the enzyme is greatly reduced, and the accumulation of reactive oxygen species endangers the plant [42]. In addition, a total of eight DEGs were identified to be involved in the biosynthesis of SOD, CAT and Pro antioxidant enzymes. Among these DEGs, one was down-regulated and associated with SOD biosynthesis, while two DEGs (one up-regulated and one down-regulated) were involved in Pro biosynthesis. Furthermore, five DEGs (three up-regulated and two down-regulated) were found to be associated with CAT biosynthesis (Figure 9, Table S5).

Excess Cd can lead to abnormal cell division and chromosome variation, resulting in a decrease in the activity of protochlorophyll reductase, which directly affects the biosynthesis of plant chlorophyll [43,44]. Previous studies have shown that when *Vigna radiata* L., *Brassica juncea,* and *Ficus parvifolia* were exposed to Cd stress, the unsaturated fatty acids on the cell membrane underwent a series of oxidation reactions, ultimately producing malondialdehyde (MDA). MDA is one of the end products of membrane lipid peroxidation, and its content can be used as one of the indicators to investigate the severity of cell stress. Its main damage leads to membrane lipid peroxidation and damages the structure of biofilm, mainly the cytoplasmic membrane, so that the structure and function of the cell membrane are damaged and the permeability of the membrane is changed, thus affecting the normal progress of a series of physiological and biochemical reactions. MDA has a negative effect on mitochondrial respiratory chain complexes and key enzyme activities in mitochondria. High levels of MDA can exacerbate cell membrane damage [45,46,47]. In our study, when *S. alba* was subjected to Cd stress, there was a significant decrease in chlorophyll a, chlorophyll b, and total chlorophyll with increasing Cd concentration, while the MDA content significantly increased (Figure 4 and Figure 5). These findings suggest that *S. alba* is sensitive to Cd, as evidenced by changes in antioxidant enzyme activity, malondialdehyde content and chlorophyll content.

Previous studies have reported that applying methyl jasmonate (MeJA) from an external source has shown a significant reduction in Cd concentration in both root and shoot tissues. Additionally, it has been observed that MeJA inhibited the expression of *AtIRT1*, *AtHMA2,* and *AtHMA4* genes in the underground part after exposure to Cd stress [48]. Jasmonic acid has the ability to decrease the expression of genes that facilitate the uptake and translocation of Cd, resulting in a decrease in Cd concentration in root cell sap and alleviating Cd stress. This ultimately enhances plant tolerance to Cd [49]. When *S. alba* was subjected to Cd stress, only eight DEGs related to the biosynthesis of jasmonic acid were identified in the roots. These DEGs mainly encoded proteins such as *JAR1*, *MYC2,* and *JAZ*, with most of them being down-regulated genes. These down-regulated DEGs could be attributed to the higher sensitivity of *S. alba* to Cd (Figure 8, Table S4).

Auxin plays a crucial role in facilitating the transport of heavy metals to the cytoplasm through the apoplast. It has the ability to initiate H^+^—ATPase, which generates an electrochemical gradient in the plasma membrane. This gradient either opens the cation channel or activates the anion transporter, resulting in the influx of metal cations [50]. Additionally, auxin influences the *DELLA* protein, indirectly regulating the balance of ROS or eliminating ROS, thereby enhancing the plant’s uptake of heavy metal elements. Our study identified 16 DEGs in the roots that are involved in the biosynthesis of auxin, primarily encoding ARF and *GH3* proteins (Figure 8, Table S4).

Cd primarily exists as Cd^2+^ ions in the soil, which can be easily absorbed by plant roots, especially in regions with root hairs. These ions accumulate in various organs of the plant due to their biological mobility [51]. Cd^2+^ is transported to the xylem through both apoplastic and symplastic pathways in the root epidermis and root cortex. Once Cd^2+^ enters the xylem, it is efficiently transported to the stems and leaves [52]. Many transport factors or genes involved in synthesis of the cell wall and lignin play an important role in the process of Cd^2+^ transport, absorption, and accumulation. ABC transporters are a class of transmembrane transporters that play a crucial role in various plant processes, including hormone transport, lipid metabolism, detoxification of toxins, and stress resistance. In *A. thaliana*, *AtABCG36* expels intracellular Cd to the outside of the cell, reducing the harm caused by Cd toxicity [53]. *AtABCB25*, located on the mitochondrial membrane, enhances the tolerance of *Arabidopsis* plants to Cd when overexpressed [54]. *AtABCC1*, *AtABCC2,* and *AtABCC3* transport Cd from the cytoplasm into vacuoles, thus minimizing the damage caused by Cd toxicity to plants [55]. In this study, we identified a total of 10 DEGs belonging to the ABC transporter family in the roots. Among them, three DEGs belonged to the *ABCA* transporter family, one DEG belonged to the *ABCB* transporter family, and five DEGs belonged to the *ABCC* transporter family (Figure 9, Table S5). Most of these genes were down-regulated, with only two up-regulated DEGs. This down-regulation may be attributed to the evident toxic effects of Cd on *S. alba*. In the phenylpropanoid biosynthesis pathway, two DEGs, *Sal02g36790L* and *Sal06g23960L*, were identified to be involved in the biosynthesis of cinnamoyl-CoA, which is responsible for secondary wall formation in plants [56]. Additionally, five DEGs (one up-regulated and four down-regulated) were found to be associated with the biosynthesis of coniferyl alcohol, which is an important component of lignin that participates in the formation of the cell wall [57].

TFs serve an important function in the regulation of gene expression in plants, particularly during plant stress responses [58]. Among these TFs, *WRKY* transcription factors have been found to have a high affinity for a specific DNA *cis*-acting element called the W-box, which is located in the promoter region. By binding to this element, *WRKY* TFs regulate the expression of downstream genes and actively participate in the response to Cd stress [59]. The overexpression of *TaWRKY70* in transgenic *Arabidopsis* was found to enhance Cd tolerance [60]. In this study, one *WRKY70* gene (*Sal11g08240L*) was predicted be down-regulated, and all *WRKY* differential genes also showed a down-regulated pattern. Additionally, studies have demonstrated that overexpression of *MYB40* leads to the expression of plant chelating enzymes and *ABCC1* and *ABCC2*, which encode the main vacuolar plant chelating protein transporters. This, in turn, increases the tolerance of plants to heavy metals [61]. In our study, one *MYB40* gene (*Sal12g05270L*) was predicted to be up-regulated (Figure 9, Table S5). These findings suggest that the identified regulatory elements may contribute to the response of *S. alba* to Cd stress.

## 5. Conclusions

In this study, *S. alba* was exposed to different concentrations of CdCl_2_ (0.25 mmol/L, 0.5 mmol/L, and 1 mmol/L) for three days. The activity and content of antioxidant enzymes and chlorophyll exhibited a decreasing trend as the concentration of heavy metals increased. RNA sequencing (RNA-seq) was employed to analyze the gene expression profiles of *S. alba* treated with CdCl_2_ (0.25 mmol/L) for 3 days. The results reveal that 37 and 542 differentially expressed genes (DEGs) were identified in leaves and roots, respectively. These DEGs were predominantly expressed in roots. The DEGs induced by Cd stress were mainly enriched in metabolic pathways, plant hormone signal transduction, and biosynthesis of secondary metabolites. In addition, we identified several transcription factors (TFs) associated with Cd tolerance, and highlighted two key DEGs involved in cell ion absorption and transporters, specially *WRKY70* (*Sal11g08240L*) and *MYB40* (*Sal12g05270L*). These findings offer valuable insights into the molecular mechanisms and physiological regulation processes of *S. alba* in response to Cd. Furthermore, they provide useful knowledge for enhancing the yield and quality of *S. alba* under Cd conditions while also serving as a theoretical foundation for food processing and medical treatment.

## Data Availability

The sequence data in raw format can be accessed at NCBI-SRA (https://www.ncbi.nlm.nih.gov/sra, accessed on 6 September 2021) using the accession number PRJNA760315.

## Supplementary Materials

The following supporting information can be downloaded at: https://www.mdpi.com/article/10.3390/genes14122224/s1, Figure S1. RT-PCR verification of the expression levels of 10 DEGs (five up-regulated and five down-regulated) determined by RNA-seq. Table S1. Cd content of heavy metal after three days of treatment. Table S2. Primers used in experiment of gene expression by RT-PCR. Table S3. Statistics on the number of up-regulated genes down-regulated genes in CKs vs. Cds and CKr vs. Cdr. Table S4. DEGs involved in plant hormone biosynthesis and plant hormone signal transduction in plants. Table S5. Antioxidant enzyme, ABC transporter protein and transcription factor in root and shoot. Table S6. phenylpropanoid biosynthesis.
