# Supplementary material for: Integrative Physiological and Transcriptome Analysis Reveals the Mechanism of Cd Tolerance in Sinapis alba
Source: Genes (Basel). 2023 Dec 16;14(12):2224. doi: 10.3390/genes14122224 (PMC10742500; doi:10.3390/genes14122224)
Supplement: Supplementary file 1 [file genes-14-02224-s001.zip › Fig. S1.qRT-PCR verification of the expression levels of 10 DEGs ( five upregulated and five downregulated ) determined by RNA-seq..pdf]

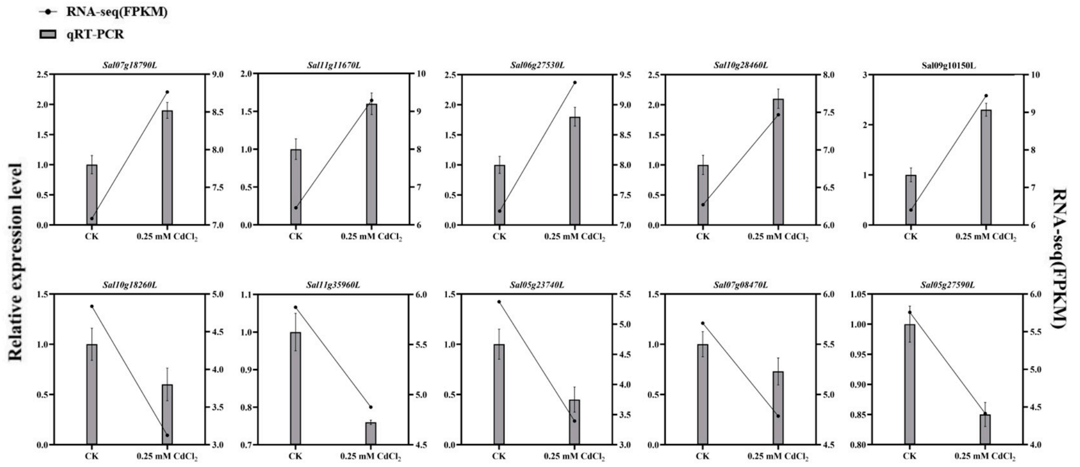

Fig. S1. qRT–PCR verification of the expression levels of 10 DEGs (five upregulated and five downregulated) determined by RNA-seq. The left y-axis represents the relative expression level, and the right y-axis represents RNA-seq (FPKM). The box with error bars indicates the qRT–PCR result, and the oblique line represents the FPKM value of gene expression.
