# Supplementary material for: Integrative Physiological and Transcriptome Analysis Reveals the Mechanism of Cd Tolerance in Sinapis alba
Source: Genes (Basel). 2023 Dec 16;14(12):2224. doi: 10.3390/genes14122224 (PMC10742500; doi:10.3390/genes14122224)
Supplement: Supplementary file 1 [file genes-14-02224-s001.zip › Table. S1. Cadmium content of heavy metal after three days of treatment.pdf]

Table. S1. Cadmium content of heavy metal after three days of treatment

Description of sample	Cadmium content (mg/kg)	Mean value (mg/kg)	Standard error
0 mM Shoots	12.73	12.80	0.34
	12.41		
	13.24		
0.25 mM Shoots	196.68	203.59	5.20
	204.85		
	209.23		
0.5 mM Shoots	233.79	233.88	3.68
	233.71		
	234.15		
1 mM Shoots	272.16	269.25	2.88
	265.32		
	270.27		
0 mM Roots	50.62	49.25	2.25
	46.08		
	51.05		
0.25 mM Roots	766.88	786.99	21.45
	764.23		
	829.86		
0.5 mM Roots	1109.06	1065.29	31.30
	1049.09		
	1037.72		
1 mM Roots	1318.42	1275.42	39.75
	1222.56		
	1285.28		
