# Supplementary material for: Integrative Physiological and Transcriptome Analysis Reveals the Mechanism of Cd Tolerance in Sinapis alba
Source: Genes (Basel). 2023 Dec 16;14(12):2224. doi: 10.3390/genes14122224 (PMC10742500; doi:10.3390/genes14122224)
Supplement: Supplementary file 1 [file genes-14-02224-s001.zip › Table. S2. Primers used in experiment of gene expression by qRT-PCR.pdf]

Table S2. Primers used in experiment of gene expression by qRT-PCR

Gene id	Forward primer	Reverse primer
actin	ACTAACTGCCTTGCTCCACT	AGCAGTGATAGAGTGGACGG
Sal07g18790L	CTGGTTCTCTTTGTGGCCGA	GTGGGGTTTGGGAATCTGGA
Sal11g11670L	ATACCGCCGCAACTTCTTCT	TAAGTCCTTGACGGCACGAG
Sal06g27530L	TACATGACCGTTGGATCGCC	CTCGCACGAAACACGTCAAG
Sal10g28460L	GTGTTGCGGGTGAGACTGTA	TTTCCACAGCAGCGAGTCAT
Sal09g10150L	AGTGGAGCACGCAAAGACAT	GAACACAACGCCAACAGTCC
Sal10g18260L	TCTCGCTAACCCTCGCATTC	CTGTGGTGGTTTTGGTTGGC
Sal11g35960L	CGTGGTATTGGTCGCGGATA	CTTCCCCCTCTTCTACACGC
Sal05g23740L	AGCCTTTCTGGATGCGACAA	CTCAAGCGGTTCCGAGACAT
Sal07g08470L	GGGAACTGGCCTTACAGAGG	AACTCCGGTGACAGAGCCTA
Sal05g27590L	AGCTCAAACCCACGAGCATT	ACTCCTGCAAAGAGGACGTG
