# Supplementary material for: Integrative Physiological and Transcriptome Analysis Reveals the Mechanism of Cd Tolerance in Sinapis alba
Source: Genes (Basel). 2023 Dec 16;14(12):2224. doi: 10.3390/genes14122224 (PMC10742500; doi:10.3390/genes14122224)
Supplement: Supplementary file 1 [file genes-14-02224-s001.zip › Table. S3. Statistics on the number of up-regulated genes down-regulated genes in CKs vs. Cds and CKr vs. Cdr.pdf]

Table S3. Statistics on the number of up-regulated genes down-regulated genes in CKs *vs*. Cds and CKr *vs*. Cdr

DEGs	CKs *vs*. Cds	CKr *vs*. Cdr
Up	19	137
Down	18	405
