# Supplementary material for: Integrative Physiological and Transcriptome Analysis Reveals the Mechanism of Cd Tolerance in Sinapis alba
Source: Genes (Basel). 2023 Dec 16;14(12):2224. doi: 10.3390/genes14122224 (PMC10742500; doi:10.3390/genes14122224)
Supplement: Supplementary file 1 [file genes-14-02224-s001.zip › Table. S4. DEGs involved in plant hormone biosynthesis and plant hormone signal transduction in plants.pdf]

Table. S4. DEGs involved in plant hormone biosynthesis and plant hormone signal transduction in plants

KEGG	ID	Symbol	transcript id	log2 (fold change) CKs *vs.* Cds	log2 (fold change) CKr *vs.* Cdr
jasmonic acid	*Sal01g00730L*	JAR1	AT2G46370.4	-0.110272738	0.733767659
	Sal12g19170L	JAZ7	AT2G34600.1	0.090035997	0.900123927
	Sal01g06990L	JAZ7	AT2G34600.1	0.000476011	-0.77380942
	Sal01g23660L	JAZ10	AT5G13220.7	-0.300425257	-1.171830343
	Sal07g26480L	JAZ1	AT1G19180.3	0.442610178	-0.662375018
	Sal09g29710L	JAZ6	AT1G72450.2	0.584632003	-0.862181821
	Sal12g04650L	JAZ10	AT5G13220.7	0.622743459	-0.675050199
	Sal08g21680L	MYC2	AT1G32640.1	0.115013376	-0.645743792
Auxin	*Sal06g32250L*	IAA30	AT3G62100.2	0.082482857	0.679328684
	Sal05g06840L	IAA24	AT1G19850.2	-0.50498866	-1.027462386
	Sal05g06840L	ARF5	AT1G19850.2	-0.50498866	-1.027462386
	Sal05g10820L	ARF8	AT5G37020.3	-0.568099568	-0.594840058
	Sal06g32370L	ARF18	AT3G61830.2	-0.347862842	-0.799949981
	Sal07g01170L	ARF11	AT2G46530.3	-0.248944525	-0.819921083
	Sal09g11650L	ARF9	AT4G23980.2	0.062523761	-1.488661374
	Sal04g26410L	GH3.5	AT4G27260.1	-0.109044918	0.736444425
	Sal09g08980L	GH3.5	AT4G27260.1	-0.214676298	0.63653736
	Sal01g23600L	GH3.12	AT5G13320.3	0.154868834	-1.384363737
	Sal03g02640L	GH3-10	AT4G03400.2	0.091998717	-0.587886643
	Sal03g24480L	GH3.12	AT5G13320.3	0.021988377	-1.497939482
	Sal12g04700L	GH3.12	AT5G13320.3	0.273473625	-1.787331645
	Sal01g00730L	GH3.11	AT2G46370.4	-0.110272738	0.733767659
	Sal06g13350L	SAUR78	AT1G72430.1	-0.100313355	0.763270316
	Sal09g03110L	SAUR49	AT4G34750.2	-0.139159115	0.778842858
