# Supplementary material for: Integrative Physiological and Transcriptome Analysis Reveals the Mechanism of Cd Tolerance in Sinapis alba
Source: Genes (Basel). 2023 Dec 16;14(12):2224. doi: 10.3390/genes14122224 (PMC10742500; doi:10.3390/genes14122224)
Supplement: Supplementary file 1 [file genes-14-02224-s001.zip › Table. S5. antioxidant enzyme, ABC transporter protein and transcription factor in root and shoot.pdf]

Table S5. Antioxidant enzyme, ABC transporter protein and transcription factor in root and shoot

KEGG	ID	Symbol	transcript id	log2(fold change) CKs *vs.* Cds	log2(fold change) CKr *vs.* Cdr
WRKY	*Sal03g24270L*	WRKY75	AT5G13080.1	0.361191997	-0.685158282
	Sal04g33120L	WRKY13	AT4G39410.1	-0.268202227	-0.778625606
	Sal06g32430L	WRKY46	AT2G46400.1	0.163911732	-0.809475739
	Sal07g01310L	WRKY46	AT2G46400.1	0.076532655	-0.628780158
	Sal09g00400L	WRKY13	AT4G39410.1	-0.226867627	-0.670501622
	Sal09g12930L	WRKY31	AT4G22070.3	0.381665586	-0.737631153
	Sal11g08240L	WRKY70	AT3G56400.1	0.284754014	-0.95994111
	Sal11g12870L	WRKY62	AT5G01900.1	-0.028512358	-0.637313551
	Sal11g27960L	WRKY51	AT5G64810.1	0.692989999	-1.04779108
	Sal12g15860L	WRKY54	AT2G40750.1	0.321408637	-0.779302514
	Sal12g15870L	WRKY55	AT2G40740.3	0.114783433	-0.967799833
	Sal01g33300L	WRKY51	AT5G64810.1	0.321471648	-0.97136528
MYB	*Sal04g31480L*	MYB32	AT4G34990.1	0.28143284	0.716844411
	Sal06g24450L	MYB65	AT3G11440.3	0.203152121	0.677810087
	Sal06g26780L	MYB108	AT3G06490.1	0.003955848	1.272103125
	Sal10g15640L	MYB56	AT5G17800.1	-0.158718689	0.700026152
	Sal11g12840L	MYB305	AT3G24310.1	0.025904163	1.000025335
	Sal11g19270L	MYB45	AT3G48920.1	0.01245687	0.734811991
	Sal12g05270L	MYB40	AT5G14340.2	0.01358962	0.669616788
	Sal12g08990L	MYB59	AT5G59780.3	0.903774003	0.670602918
	Sal02g06750L	MYB122	AT1G74080.1	0.087399076	-0.685231714
	Sal02g06990L	MYB95	AT1G74430.2	0.183995267	-1.03068712
	Sal04g19620L	MYB34	AT5G60890.1	-0.140085505	-1.212893513
	Sal05g06230L	MYB51	AT1G18570.1	0.568165198	-0.869440886
	Sal09g22460L	MYB94	AT3G47600.2	0.729727138	-0.998551314
	Sal10g21110L	MYB88	AT2G02820.3	-0.106929796	-0.615358254
	Sal12g24330L	MYB81	AT2G26960.1	-0.138874571	-0.798983795
	Sal01g13550L	MYB73	AT4G37260.1	-0.179071643	0.599154329
bHLH	*Sal06g27630L*	bHLH96	AT1G72210.2	0.033846386	0.640056287
	Sal03g11130L	bHLH	AT5G51780.2	-0.026235253	-0.602038113
	Sal04g06080L	bHLH92	AT5G43650.2	0.291992944	-1.083547325
	Sal07g01190L	bHLH017	AT2G46510.1	0.329614275	-0.67708773
	Sal12g22990L	bHLH029	AT2G28160.2	0.179955981	-0.841626369
	Sal01g34110L	bHLH093	AT5G65640.2	0.144436609	0.623194616
CAT	*Sal04g24250L*	CAT1	AT4G21120.2	0.630458451	0.651238664
	Sal09g24930L	CAT7	AT1G50090.1	0.035580421	0.955087571
	Sal05g07150L	CAT3	AT1G20620.7	0.046931768	-0.70896001

	Sal08g06480L	CAT2	AT1G10070.2	0.3789437	-0.714217328
	Sal08g13780L	CAT3	AT1G20620.7	0.166475978	-0.994906842
Pro	*Sal05g07420L*	PRO25	AT1G21250.1	-0.166508468	1.051896764
	Sal02g33330L	PRO2	AT2G16650.2	-0.165178409	-0.695601386
SOD	*Sal02g03110L*	SOD1	AT1G08830.2	-0.42364719	-0.642098622
ABCA	*Sal06g23290L*	ABCA4	AT3G47750.1	0.23292205	1.118856045
	Sal09g22530L	ABCA7	AT3G47780.1	-0.049055171	0.676516227
	Sal07g07850L	ABCA8	AT3G47790.2	0.039290479	-1.228588422
ABCB	*Sal08g01080L*	ABCB11	AT1G02520.2	0.128634795	-0.837879287
	Sal09g10000L	ABCB2	AT4G25960.1	-0.268731066	-0.81092096
ABCC	*Sal02g24130L*	ABCC7	AT3G13100.2	0.706111816	-0.665304297
	Sal05g10850L	ABCC1	AT1G30400.2	0.037519924	-0.659901318
	Sal07g18830L	ABCC2	AT2G34660.3	0.088614868	-0.682198003
	Sal08g27560L	ABCC4	AT2G47800.1	0.638220498	-0.663210873
	Sal02g15450L	ABCC9	AT3G60160.3	0.742256547	-1.35929781
