# Supplementary material for: Integrative Physiological and Transcriptome Analysis Reveals the Mechanism of Cd Tolerance in Sinapis alba
Source: Genes (Basel). 2023 Dec 16;14(12):2224. doi: 10.3390/genes14122224 (PMC10742500; doi:10.3390/genes14122224)
Supplement: Supplementary file 1 [file genes-14-02224-s001.zip › Table. S6 Phenylpropanoid biosynthesis.pdf]

Table. S6 Phenylpropanoid biosynthesis

KEGG	ID	Symbol	transcript id	log2(fold change) CKs *vs.* Cds	log2(fold change) CKr *vs.* Cdr
Cinnamoyl-CoA	*Sal02g36790L*	HCT	AT5G48930.1	0.113068792	0.018237949
	Sal06g23960L	DCF	AT3G48720.1	-0.244067217	-0.027699621
Coniferyl alcohol	*Sal01g06920L*	EDA28, MEE23, AtBBE-like15	AT2G34790.1	0.485216119	-0.127746262
	Sal04g20680L	UGT72E1	AT3G50740.1	-0.205431936	-0.337378813
	Sal04g25500L	AtDIR6, DIR6	AT4G23690.1	0.053372702	-0.39603638
	Sal05g11550L	AtPCBER1, PCBER1	AT4G39230.1	0.136355399	0.01044705
	Sal07g18740L	EDA28, MEE23, AtBBE-like15	AT2G34790.1	0.168779034	-0.300099806
